# 

**Published:** 1887-12

**Authors:** 


### CONTENTS.

COMMUNICATIONS.

Pyorrhoea Alveolaris with Practical Cases.................................. 577

PROCEEDINGS.

Ninth International Medical Congress, Washington, D. C., Sept., 1887. 585

EDITORIAL.

SALMAGUNDI........................................................................ 629

